# Relationship between sedentary behavior and endothelial dysfunction in a cross-sectional study in China

**DOI:** 10.3389/fcvm.2023.1148353

**Published:** 2023-08-09

**Authors:** Ping-ting Yang, Sai-qi Yang, Yong-mei He, Jian-gang Wang, Yue-xiang Qin, Ya-qin Wang, Ying Li

**Affiliations:** ^1^ Department of Health Management, The Third Xiangya Hospital, Central South University, Changsha, China; ^2^ Department of Health Management, Aerospace Center Hospital, Beijing, China

**Keywords:** sedentary behavior, endothelial dysfunction, FMD, work hours, check-up population

## Abstract

Sedentary behavior is a risk factor for several diseases, and previous studies have mostly reported the effects of acute sedentary behavior on vascular endothelial function. Data on the relationship between sedentary lifestyle habits and vascular function in large sample populations are lacking. Therefore, the aim of this study was to assess the correlation between self-reported sedentary behavior and peripheral vascular function in a check-up population from real-world data.

**Methods:**

We recruited 13,220 participants from two health management centers of general tertiary hospitals located in northern and southern China between 2017 and 2021. All participants had undergone both questionnaires and brachial artery flow-mediated dilation (FMD) measurements.

**Results:**

In total, 3,205 participants with FMD ≤ 5.0% were identified to have endothelial dysfunction. In a multivariable regression model including lifestyle habits such as sedentary behavior and cardiovascular risk factors, taking leisure sedentary time <2 h/day as a reference, the risk of vascular endothelial dysfunction gradually increased with time: 2–4 h/day (OR* *= 1.182, 95% CI: 1.058–1.321, *P *= 0.003), 4–6 h/day (OR* *= 1.248, 95% CI: 1.100–1.414, *P *= 0.001) and >6 h/day (OR* *= 1.618, 95% CI: 1.403–1.866, *P *< 0.001).

**Conclusion:**

Longer leisure sedentary time is associated with a higher prevalence of vascular endothelial dysfunction. These findings suggest that leisure sedentary behavior is a risk factor for the occurrence of vascular endothelial dysfunction in the Chinese check-up population.

## 1. Introduction

Excessive sedentary behavior is independently associated with adverse health indicators and various chronic diseases, such as peripheral and central vascular disease (1), coronary artery disease (2), type 2 diabetes mellitus (3), depression (4), stroke (5, 6), cancer (7) and mortality (8, 9). A large proportion of the population spends most of their waking hours in a sedentary position, despite these known health risks (10). Previous studies of sedentary behavior have found that people self-report sitting for 180–480 min per day (11, 12). Sedentary behavior compresses the lower limbs and reduces venous return, leading to transient vascular endothelial dysfunction (13), which is a precursor to the development of atherosclerosis and is one of the early detectable indicators of vascular health that can be used to screen and assess the risk of cardiovascular disease (CVD) in the population, and interventions for endothelial dysfunction can potentially prevent the future risk of CVD in healthy people (14).

Flow-mediated vasodilatation (FMD) of the brachial artery has become the most widely used technique to evaluate endothelial function. The technique quantifies the ability of larger conduit arteries to dilate in response to reactive hyperemia after a brief (5 min) suprasystolic occlusion of the brachial artery using a blood pressure cuff (14). The lower the value of the FMD, the worse the function of the vascular endothelium. In this study, FMD <5% was used as a cutoff value to distinguish whether there was endothelial dysfunction. As previous studies have been conducted mainly in small sample populations and most of them studied the effect of acute sedentary time on vascular endothelial function in the short term, data on the relationship between sedentary lifestyle habits and vascular function in large sample populations are lacking. Therefore, the aim of this study was to evaluate the correlation between self-reported lifestyle habits such as sedentary time and work hours and peripheral vascular function in a healthy population from real-world data. The results of this study contribute to the current understanding of persistence as a potentially modifiable risk factor for CVD and help identify new targets for interventions to prevent the risk of cardiometabolic disease.

## 2. Subjects and methods

**Figure 1:**
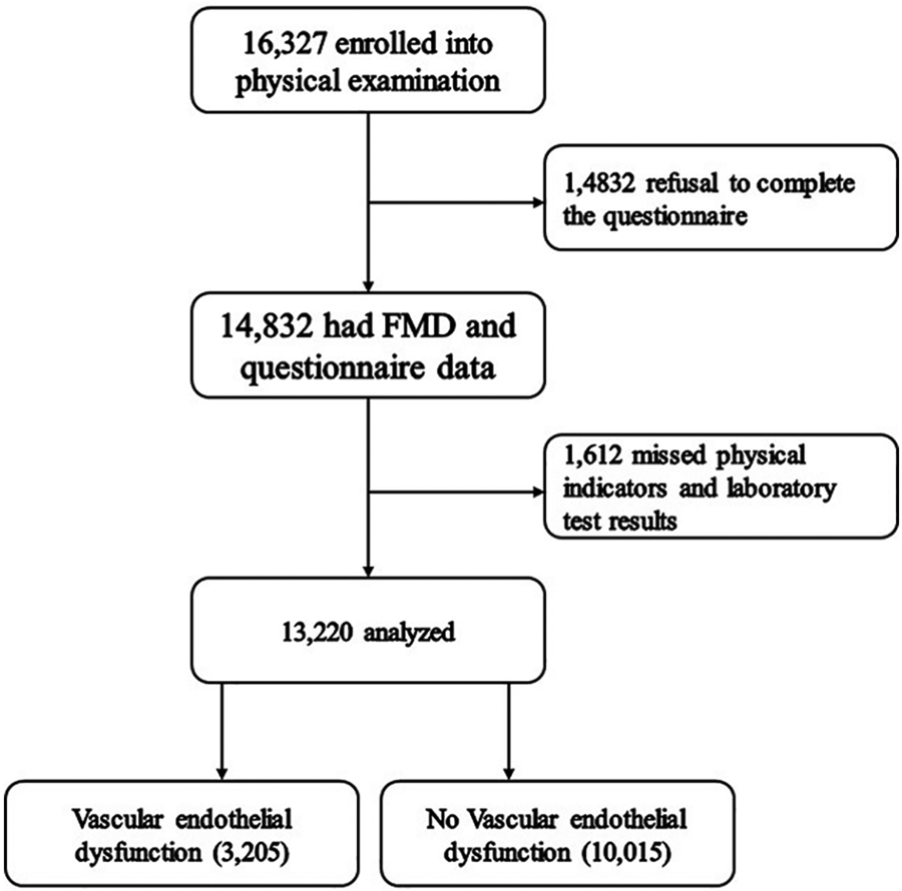
Flowchart of this study.

The participants were recruited from two departments of health management of general tertiary hospitals located in northern and southern China. Participants who met the following criteria were included in the study: (1) aged ≥18; (2) volunteered to participate in this study; (3) underwent vascular endothelial function tests; and (4) could understand and complete questionnaires. Those who experienced major physical and mental illnesses, including severe liver and kidney dysfunction, malignant tumors, and acute disease conditions, were excluded. A total of 13,220 individuals were included in this study from January 1, 2017, to December 31, 2021. Enrolled participants were divided into groups with and without vascular endothelial dysfunction based on the results of vascular endothelial function tests. The physical examination protocol and the consent form for this study were reviewed by the institutional review boards of both hospitals and approved by the independent ethics committee of the Third Xiangya Hospital of Central South University (No. 2018-S389). Written informed consent was provided in this study following the general recommendations of the Declaration of Helsinki. The study flow is shown in Figure 1.

### 2.1. Individual characteristics and lifestyle

Physical examination was performed to obtain information on height, weight, waist circumference (WC) and blood pressure (BP) and to calculate body mass index (BMI). The above indicators are averaged twice to exclude measurement errors. Information on individual health habits, disease history and medication use history are obtained by completing the National Physical Examination Questionnaire (15) under trained nurses. All information was collected through self-report. Participants were asked whether they smoked, whether they quit smoking and whether they were passive smokers. Participants were asked if they drank alcohol and if they had quit. Participants were asked how many hours of sedentary behaviors in leisure time they had on a typical day, such as watching TV, using computers and driving a car (16). Leisure sedentary time was defined as <2 h/day, 2–4 h/day, 4–6 h/day and >6 h/day. Sleep duration was defined as <5 h/day, 5–7 h/day and >7 h/day.

### 2.2. Measurements of FMD

FMD was measured according to published guidelines (14) using a high-resolution linear arterial transducer with computer-aided analysis software (UNEXEF18G; Unex Co. Ltd., Nagoya, Japan) and an automated margin detection system to measure the brachial artery diameter. All vascular function assessments were performed in a quiet, dim-lit, temperature-controlled (22°C–25°C) room. All examinations were performed by an experienced ultrasonographer between 8am and 12pm. The patient was instructed to stop taking medication that interferes with vascular function 24 h prior to the examination and to abstain from coffee and tea and from smoking on the day of the examination. The sphygmomanometer cuff is then placed on the upper arm with the lower edge of the cuff 2–3 cm above the transverse elbow line, the cuff is inflated to a level greater than 50 mmHg systolic pressure, and the blood flow is blocked for 5 min. The subject is asked to be quiet and to remain in the above position during the blockade. The internal diameter of the brachial artery is measured 45–60 s after the cuff has been deflated. FMD = [(maximum brachial artery diameter—resting brachial artery diameter)/resting brachial artery diameter] × 100%. This method has been validated previously (17).

### 2.3. Laboratory measurements

All laboratory tests were performed by certified laboratory physicians from the central laboratory department of the hospital using standard protocols. The biochemical tests included venous blood fasting glucose, total cholesterol (TC), triglycerides (TG), low-density lipoprotein cholesterol (LDL-C), high-density lipoprotein cholesterol (HDL-C), creatinine (Cr) and uric acid (UA) measured after an early morning fast of 12 h.

### 2.4. Definition of chronic disease

Hypertension was defined as self-reported hypertension diagnosed by a physician, self-reported regular use of antihypertensive medications, or systolic/diastolic blood pressure at recruitment ≥140/90 mmHg (18).

Dyslipidemia was defined as meeting any of the following criteria: (1) TC ≥ 6.22 mmol/L; (2) LDL-C ≥ 4.14 mmol/L; (3) HDL-C < 1.04 mmol/L; (4) TG ≥ 2.26 mmol/L; (5) use of antihyperlipidemic medications; and 6) self-reported dyslipidemia diagnosed by a physician (19).

Diabetes mellitus was defined as self-reported diabetes diagnosed by a physician, self-reported regular use of antidiabetic medications, or fasting glucose at recruitment ≥7.0 mmol/L (20).

CVD was defined as a patient who had been diagnosed with acute coronary syndrome, had stenting or thrombolytic therapy, or had a coronary CTA or coronary angiogram to confirm the diagnosis of coronary artery disease (21).

### 2.5. Statistical analyses

All collected data were analyzed by Statistical Package for Social Sciences (SPSS Inc., Chicago, IL, version 22.0 for Windows). Continuous variables are shown as the means ± standard deviations (SD), and categorical variables are reported as percentages (%) and numbers (n). Differences in baseline characteristics, lifestyle, blood test results and chronic disease between patients with and without vascular endothelial dysfunction were compared using independent samples *t*-tests and *χ*^2^ tests. Multivariable logistic regression analysis was used to assess the association between sedentary behavior, lifestyle, chronic disease and vascular function to adjust for confounding. Model 1 included only individual characteristics; model 2 included individual characteristics and dietary habits; and model 3 was a full model adding chronic disease to the variables included in model 2. A predefined alpha of 0.05 was used. All *P*-values were 2-tailed.

## 3. Results

**Figure 2:**
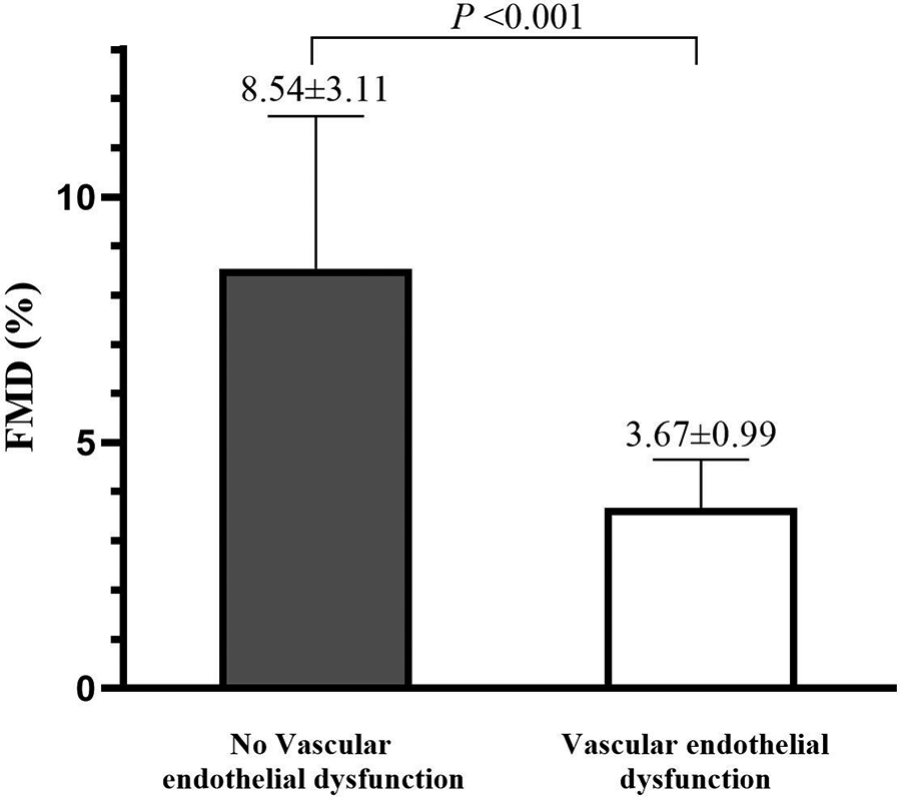
The difference of FMD (%) in the no vascular endothelial dysfunction or vascular endothelial dysfunction group. FMD, Flow-mediated dilation.

The characteristics of the participants are summarized in Tables 1, 2. A total of 13,220 subjects were enrolled in this study. The mean age of the participants was 48.94 years old, and 76.61% of the participants were male. Self-reported current smokers and current drinkers accounted for 35.85% and 51.82% of the total, respectively. The highest percentage of sedentary time was 2–4 h (40.16%), and the highest percentage of sleep duration was 5–7 h (62.32%). The percentages of participants diagnosed with hypertension, diabetes, dyslipidemia and cardiovascular disease were 23.79%, 14.61%, 38.22% and 1.79%, respectively. The study included 3,205 vascular endothelial dysfunction patients and 10,015 individuals without vascular endothelial dysfunction. The prevalence of vascular endothelial dysfunction was 24.24% in the Chinese health examination population. There were statistical differences between the two groups on all indicators except DBP, sleep duration, LDL-C and HDL-C. Participants with vascular endothelial dysfunction had a significantly lower FMD (3.67 ± 0.99%) than those with vascular endothelial dysfunction (8.54 ± 3.11%), as presented in Figure 2.

**Table 1:** Individual characteristics of all participants with and without vascular endothelial dysfunction.

Characteristics (mean ± SD)	All participants (*N* = 13,220)	Vascular endothelial dysfunction (*n* = 3,205)	No vascular endothelial dysfunction (*n* = 10,015)	*t/x* ^2^	*P* ^2^
Age (years)	48.94 ± 9.96	50.05 ± 9.98	48.59 ± 9.93	7.248	<0.001
Male sex % (*n*)	76.61 (10,128)	80.47 (2,579)	75.38 (7,549)	35.121	<0.001
BMI (kg/m^2^)	25.31 ± 3.28	25.61 ± 3.32	25.22 ± 3.26	5.982	<0.001
Waist (cm)	86.60 ± 9.56	88.07 ± 9.49	86.13 ± 9.53	10.026	<0.001
SBP (mmHg)	127.64 ± 17.25	128.89 ± 17.21	127.24 ± 17.23	4.727	<0.001
DBP (mmHg)	80.30 ± 11.98	80.39 ± 12.01	80.27 ± 11.96	0.483	0.629
Baseline brachial artery diameter (mm)	4.25 ± 0.67	4.50 ± 0.67	4.17 ± 0.65	25.005	<0.001
Max brachial artery diameter (mm)	4.56 ± 0.69	4.66 ± 0.69	4.52 ± 0.69	10.270	<0.001
Alcohol consumption % (*n*)				15.176	0.001
None	51.82 (6,851)	50.73 (1,626)	52.17 (5,225)		
Yes	45.79 (6,054)	45.99 (1,474)	45.73 (4,580)		
Abstinent from alcohol	2.38 (315)	3.28 (105)	2.10 (210)		
Smoking % (*n*)				25.604	<0.001
Nonsmoker	53.85 (7,119)	51.01 (1,635)	54.76 (5,484)		
Ex-smoker	5.40 (714)	5.80 (186)	5.27 (528)		
Passive-smoker	4.89 (647)	4.09 (131)	5.15 (516)		
Current	35.85 (4,740)	39.10 (1,253)	34.82 (3,487)		
Sedentary time % (*n*)				40.191	<0.001
<2 h/day	24.21 (3,200)	21.25 (681)	25.15 (2,519)		
2–4 h/day	40.16 (5,309)	40.12 (1,286)	40.17 (4,023)		
4–6 h/day	22.72 (3,004)	22.96 (736)	22.65 (2,268)		
>6 h/day	12.91 (1,707)	15.66 (502)	12.03 (1,205)		
Sleep duration % (*n*)				5.114	0.078
<5 h/day	10.72 (1,417)	11.73 (376)	10.39 (1,041)		
5–7 h/day	62.32 (8,239)	62.12 (1,991)	62.39 (6,248)		
>7 h/day	26.96 (3,564)	26.15 (838)	27.22 (2,726)		

SD, standard deviation; BMI, body mass index; SBP, systolic blood pressure; DBP.

**Table 2:** Lab values and comorbidities of all participants with and without vascular endothelial dysfunction.

Characteristics (mean ± SD)	All participants (*N* = 13,220)	Vascular endothelial dysfunction (*n* = 3,205)	No vascular endothelial dysfunction (*n* = 10,015)	*t/x* ^2^	*P* ^2^
FSB (mmol/L)	5.66 ± 1.59	5.91 ± 1.82	5.58 ± 1.50	9.310	<0.001
SCr (mmol/L)	75.02 ± 19.91	76.64 ± 25.11	74.50 ± 17.90	4.467	<0.001
BUN (mmol/L)	4.98 ± 1.31	5.12 ± 1.35	4.94 ± 1.29	7.091	<0.001
UA (mmol/L)	351.91 ± 92.69	364.28 ± 89.49	347.96 ± 93.34	8.891	<0.001
TC (mmol/L)	5.19 ± 0.98	5.26 ± 1.03	5.17 ± 0.96	4.770	<0.001
TG (mmol/L)	2.17 ± 1.86	2.30 ± 2.11	2.12 ± 1.77	4.428	<0.001
HDL-C (mmol/L)	1.35 ± 0.34	1.36 ± 0.34	1.35 ± 0.34	0.090	0.928
LDL-C (mmol/L)	2.86 ± 0.87	2.88 ± 0.91	2.86 ± 0.86	1.265	0.206
Hypertension % (*n*)	23.79 (3,145)	38.41 (1,231)	19.11 (1,914)	498.704	<0.001
Dyslipidemia % (*n*)	38.22 (5,053)	41.72 (1,337)	37.10 (3,716)	21.869	<0.001
Diabetes mellitus % (*n*)	14.61 (1,932)	19.81 (635)	12.95 (1,297)	91.625	<0.001
CVD % (*n*)	1.79 (237)	2.96 (95)	1.42 (142)	32.972	<0.001

SD, standard deviation; FSB, fasting serum glucose; SCr, serum creatinine; BUN, blood urea nitrogen; UA, uric acid; TC, total cholesterol; TG, triglycerides; HDL-C, high-density lipoprotein cholesterol; LDL-C, low-density lipoprotein cholesterol.

**Figure 3:**
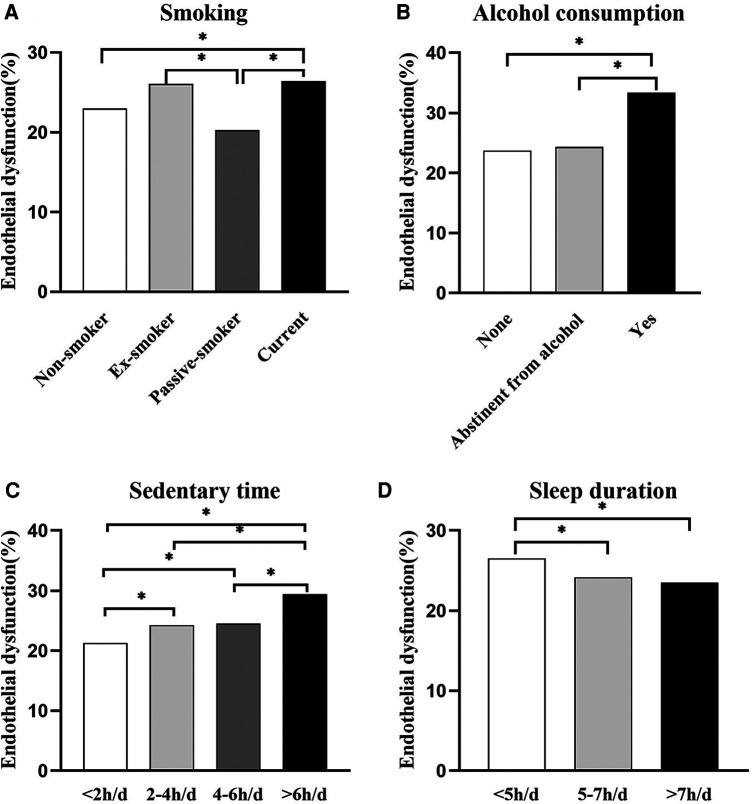
Bar graph showing the relationship between the prevalence of vascular endothelial dysfunction and various lifestyle habits. (**A**) People with different smoking habits, (**B**) people with different alcohol consumption habits, (**C**) people with different sedentary time, (**D**) people with different sleep duration. The error bars indicate the standard deviation. *P < 0.05.

Figure 3 illustrates the relationship between vascular endothelial dysfunction and lifestyle in the whole population. Current smokers had the most vascular endothelial dysfunction, and the difference was statistically significant compared to nonsmokers and passive smokers. Vascular endothelial dysfunction was greatest among those who drank alcohol and was significantly different from both the nondrinking and abstinent alcohol populations. The highest percentage of vascular endothelial dysfunction was found in the >6 h/day group among those with different sedentary times, and the differences were statistically significant between the other groups, except for no differences between the 2–4 h/day and 4–6 h/day groups. Among the different sleep durations, the highest percentage of people with vascular endothelial dysfunction was found in the <5 h/day group, with significant differences compared to both the 5–7 h/day group and the >7 h/day group.

Logistic regression analysis was used to estimate the odds ratio (OR) and 95% confidence interval (CI) for the prevalence of vascular endothelial dysfunction in the population, as shown in Table 3. Sedentary time was associated with the prevalence of vascular endothelial dysfunction in model 1 adjusted for age, sex, BMI and baseline brachial artery diameter and in model 2 further adjusted for other lifestyles (*P* < 0.001). Model 3, which adjusted for SBP, FBS, UA, TC, TG, HDL-C, LDL-C, hypertension, diabetes mellitus, dyslipidemia and CVD indicators on top of model 2, found that taking leisure sedentary time <2 h/day as a reference, the risk of vascular endothelial dysfunction gradually increased with time: 2–4 h/day (OR* *= 1.182, 95% CI: 1.058–1.321, *P *= 0.003), 4–6 h/day (OR* *= 1.248, 95% CI: 1.100–1.414, *P *= 0.001) and >6 h/day (OR* *= 1.618, 95% CI: 1.403–1.866, *P *< 0.001).

**Table 3:** Correlation of vascular endothelial function with sedentary time and work hours (*N* = 13,220).

Variables	Model 1		Model 2		Model 3	
	OR (95% CI)	*P*	OR (95% CI)	P	OR (95% CI)	*P*
Sedentary time						
<2 h/day	Ref.		Ref.		Ref.	
2–4 h/day	1.183 (1.062, 1.319)	0.002	1.192 (1.069, 1.329)	0.002	1.182 (1.058, 1.321)	0.003
4–6 h/day	1.222 (1.082, 1.381)	0.001	1.239 (1.096, 1.401)	0.001	1.248 (1.100, 1.414)	0.001
>6 h/day	1.566 (1.363, 1.798)	<0.001	1.580 (1.374, 1.817)	<0.001	1.618 (1.403, 1.866)	<0.001

Model 1: adjusted for age, sex, BMI, and baseline brachial artery diameter.

Model 2: adjusted for age, sex, BMI, baseline brachial artery diameter, alcohol consumption and smoking and sleep duration.

Model 3: adjusted for age, sex, BMI, baseline brachial artery diameter, alcohol consumption, smoking, sleep duration, SBP, FBS, UA, TC, TG, HDL-C, LDL-C, hypertension, diabetes mellitus, dyslipidemia and CVD.

The relationship between vascular endothelial dysfunction and individual characteristics, sedentary time and common risk factors is shown in Supplementary Table S1 and Figure S1. Increased age (*P* < 0.001) and BMI (*P* = 0.014) were also risk factors for endothelial dysfunction. Smokers (*P* = 0.017) showed a significantly higher risk than nonsmokers. Hypertension (*P* < 0.001) and coronary heart disease (*P* = 0.003) were risk factors for the development of vascular endothelial dysfunction compared with those without these diseases.

## 4. Discussion

To the best of our knowledge, this is the first study to examine the relationship between leisure sedentary time and vascular endothelial function in a large sample of people. This study found that leisure sedentary time was positively associated with the occurrence of vascular endothelial dysfunction after other confounders were controlled. No significant associations were observed for sleep duration. Therefore, reducing leisure sedentary behavior can effectively improve endothelial dysfunction.

In this study, FMD <5% was used as a cutoff value to distinguish whether there was endothelial dysfunction. FMD values defining vascular endothelial dysfunction have been reported differently in different studies, ranging from 3.7%–11.3% (22–29). However, in clinical work, 5% (28) and 6% (29)are considered to be more appropriate cutoff values for people with potential endothelial dysfunction and are also the most widely used in screening large samples of people. Previous studies have shown that sustained periods of reduced sedentary behavior can improve peripheral vascular function and reduce the incidence of impaired vascular function (30). In addition, reducing sedentary time by 100 min/day and increasing physical activity by 20 min/day can improve vascular endothelial function (12). In sedentary older patients, land walking can also effectively improve endothelial function (31).

In this study, the longer the self-reported leisure sedentary time in a day, the higher the risk of vascular endothelial dysfunction. This is similar to the results of a previous study, a cross-sectional study including 98 healthy participants, which found that sedentary breaks and total time spent in sedentary bouts of more than one hour were predictors of absolute FMD (32). In a study that included 24 patients at increased cardiovascular risk, FMD was found to be significantly greater in patients who reduced sedentary time than in those with no change in sedentary time, suggesting that long-term reductions in sedentary behavior may improve peripheral vascular function (30). This also occurred in patients with diabetes. The study found that interrupting sedentary activity every 30 min instead of 60 min significantly increased FMD over a 7-hour period compared to sitting. Therefore, more frequent, shorter breaks may be more beneficial than longer, less frequent breaks for the vascular health of patients with diabetes (33). Other studies have measured endothelial function in 28 habitually active participants at baseline, 14 days after reducing steps and 14 days after resuming habitual activity and found that while a decrease in endothelial function was observed after short-term physical activity, this could be reversed after resuming habitual activity (34). The dose‒response relationship between prolonged sitting and vascular function was investigated by meta-analysis in healthy individuals and patients with metabolic disorders, and it was found that prolonged sitting progressively impairs lower extremity vascular function, while no similar trend was observed for upper extremity vascular function (13). Subgroup analysis showed that prolonged sitting had a negative impact on healthy individuals, but this finding was not observed in the metabolic disorder population (35).

Leisure sedentary behavior is any waking behavior with an energy expenditure less than 1.5 metabolic equivalents in a sitting, reclining, or lying posture, including sedentary television watching, computer use, and driving, excluding sitting at work (36). The main cause of vascular endothelial dysfunction due to sedentary sitting is the reduction in muscle activity and the subsequent reduction in energy demand leading to a decrease in peripheral blood flow, causing a decrease in shear stress, which in turn increases vasoconstriction, leading to vascular dysfunction (37). There are many possible explanations for the difference between sedentary leisure and occupational sitting. First, leisure sedentary may replace physical activity during leisure time. Second, leisure sedentary tends to be more of an uninterrupted sedentary behavior, whereas occupational sitting can be more easily interrupted. Third, leisure sedentary is more associated with increased intake of a high calorie, high sugar diet, while postprandial blood glucose, insulin and triglyceride levels can rise sharply, also leading to a mechanism of impaired vascular function (37).

Several limitations of our study need to be considered. First, sedentary behavior was obtained by self-report rather than from objectively measured data, which is still lacking in accuracy, but real-world data from a large sample better reflect the relationship between self-perceptions and objectively measured outcomes. Second, this study population was representative of two general hospital health management facilities in southern and northern China, but the findings may not be generalizable to other populations more broadly. Third, although we tried to control for known potential confounding variables, there may be other confounding factors that were not measured or not available. Fourth, due to the use of specific questionnaires to collect information, there is a lack of comprehensive information in this study. There was no information on the type of work and unable to distinguish between specific types of hypertension such as White coat hypertension and masked hypertension, which would have affected the measurement results. Finally, because of the cross-sectional nature of this study, it is not possible to infer a causal relationship between sedentary behavior and endothelial dysfunction.

## 5. Conclusion

In this sample of the Chinese medical examination population, we found that leisure sedentary behavior was positively associated with vascular endothelial dysfunction. Future research needs to quantify and standardize sedentary behavior, and subdivide sedentary behavior, such as leisure sedentary behavior and work sedentary behavior, to more accurately explore the impact of life and work behavior on vascular function.

## Data Availability

The raw data supporting the conclusions of this article will be made available by the authors, without undue reservation.

## Supplementary material

Supplementary Figure S1 Forest plots of odds ratios and 95% confidence intervals of the prevalence of endothelial dysfunction in various lifestyle habit subgroups.
