# Supplementary material for: Relationship between sedentary behavior and endothelial dysfunction in a cross-sectional study in China
Source: Front Cardiovasc Med. 2023 Aug 9;10:1148353. doi: 10.3389/fcvm.2023.1148353 (PMC10445148; doi:10.3389/fcvm.2023.1148353)
Supplement: Supplementary file 1 [file Table1.docx]

**Table S1. Multilevel logistic regression analysis of the relationship between vascular endothelial dysfunction and individual characteristics, sedentary time and common risk factors (N = 1,3220)**

| **Variables** | **β** | **OR (95%CI)** | **P** |
| --- | --- | --- | --- |
| Sex |  |  |  |
| Male |  | Ref. |  |
| Female | -0.109 | 0.897 (0.797, 1.010) | 0.072 |
| age | 0.015 | 1.015 (1.010, 1.019) | <0.001 |
| BMI | 0.018 | 1.018 (1.004, 1.033) | 0.014 |
| Baseline brachial artery diameter | 0.745 | 2.107 (1.969, 2.256) | <0.001 |
| Alcohol consumption | -0.136 | 0.873 (0.805, 0.947) | 0.001 |
| Smoking |  |  |  |
| Nonsmoker |  | Ref. |  |
| Ex-smoker | -0.015 | 0.985 (0.816, 1.189) | 0.873 |
| Passive-smoker | -0.170 | 0.844 (0.684, 1.042) | 0.115 |
| Current | 0.117 | 1.124 (1.021, 1.236) | 0.017 |
| Sedentary time |  |  |  |
| <2 |  | Ref. |  |
| 2-4 | 0.168 | 1.182 (1.058, 1.321) | 0.003 |
| 4-6 | 0.221 | 1.248(1.100, 1.414) | 0.001 |
| >6 | 0.481 | 1.618 (1.403, 1.866) | <0.001 |
| Sleep duration |  |  |  |
| <5 |  | Ref. |  |
| 5-7 | -0.098 | 0.907 (0.792, 1.039) | 0.159 |
| >7 | -0.145 | 0.865 (0.745, 1.005) | 0.058 |
| Hypertension | 0.822 | 2.275 (2.075, 2.495) | <0.001 |
| Diabetes mellitus | 0.140 | 1.150 (0.996, 1.329) | 0.057 |
| CVD | 0.423 | 1.526 (1.152, 2.023) | 0.003 |
| Dyslipidemia | -0.005 | 0.995 (0.888, 1.115) | 0.927 |
| SBP | 0.001 | 1.001 (0.999, 1.004) | 0.327 |
| FBS | 0.056 | 1.057 (1.024, 1.091) | 0.001 |
| UA | 0.002 | 1.002 (1.001, 1.002) | <0.001 |
| TC | 0.037 | 1.038 (0.874, 1.233) | 0.673 |
| TG | 0.010 | 1.010 (0.949, 1.075) | 0.759 |
| HDL-C | 0.497 | 1.644 (1.335, 2.024) | <0.001 |
| LDL-C | -0.005 | 0.955(0.995, 0.836) | 1.184 |
