# Supplementary figures and images for: Relationship between sedentary behavior and endothelial dysfunction in a cross-sectional study in China
Source: Front Cardiovasc Med. 2023 Aug 9;10:1148353. doi: 10.3389/fcvm.2023.1148353 (PMC10445148; doi:10.3389/fcvm.2023.1148353)

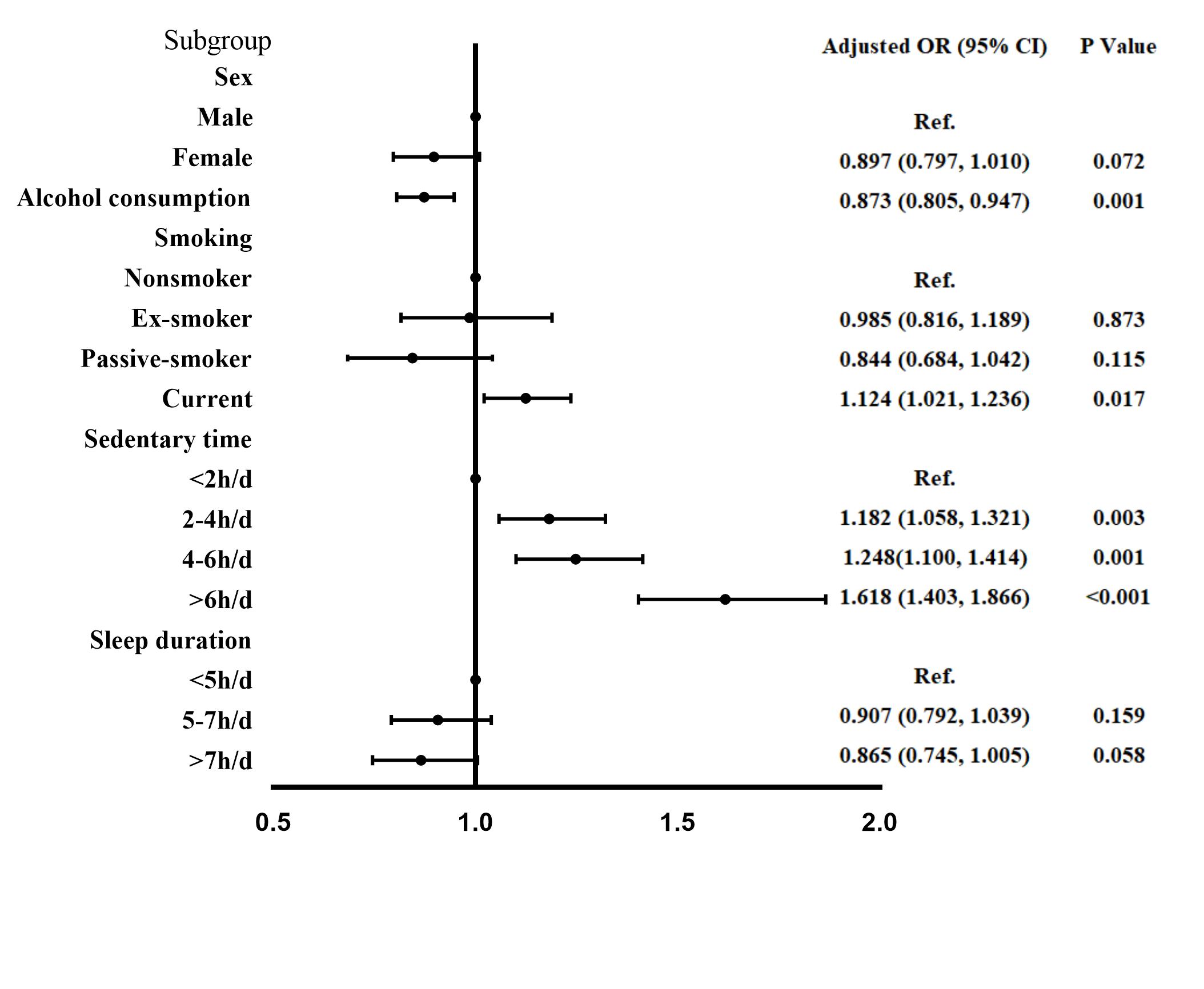

Supplement: Supplementary Figure S1 — Forest plots of odds ratios and 95% confidence intervals of the prevalence of endothelial dysfunction in various lifestyle habit subgroups. [file Image1.jpg]
